# Early and gender-specific differences in spinal cord mitochondrial function and oxidative stress markers in a mouse model of ALS

**DOI:** 10.1186/s40478-015-0271-6

**Published:** 2016-01-13

**Authors:** Daniel Cacabelos, Omar Ramírez-Núñez, Ana Belén Granado-Serrano, Pascual Torres, Victòria Ayala, Victoria Moiseeva, Mònica Povedano, Isidre Ferrer, Reinald Pamplona, Manuel Portero-Otin, Jordi Boada

**Affiliations:** The Metabolic Pathophysiology Research Group, Deparment Experimental Medicine, University of Lleida-Institute for Research in Biomedicine of Lleida (UdL- IRBLleida), Avda Rovira Roure, 80, E-25198 Lleida, Spain; The Neurology Service, Hospital Universitari de Bellvitge, L’Hospitalet de Llobregat, c/La Feixa Llarga, S/N 08908 Hospitalet de Llobregat, Barcelona, Spain; Institut de Neuropatologia, Hospital Universitari de Bellvitge - IDIBELL, Universitat de Barcelona, and CIBERNED (Centro de Investigación Biomédica en Red de Enfermedades Neurodegenerativas), Instituto Carlos III, Spanish Ministry of Health, Spain. L’Hospitalet de Llobregat, c/La Feixa Llarga, S/N 08908 Hospitalet de Llobregat, Barcelona, Spain

**Keywords:** Motor neuron, Complex I, Respirometry, Fatty acid composition, Oxidative damage, Estrogens

## Abstract

**Introduction:**

Amyotrophic lateral sclerosis (ALS) is a motor neuron disease with a gender bias towards major prevalence in male individuals. Several data suggest the involvement of oxidative stress and mitochondrial dysfunction in its pathogenesis, though differences between genders have not been evaluated. For this reason, we analysed features of mitochondrial oxidative metabolism, as well as mitochondrial chain complex enzyme activities and protein expression, lipid profile, and protein oxidative stress markers, in the Cu,Zn superoxide dismutase with the G93A mutation (hSOD1-G93A)- transgenic mice and Neuro2A(N2A) cells overexpressing hSOD1-G93A.

**Results and Conclusions:**

Our results show that overexpression of hSOD1-G93A in transgenic mice decreased efficiency of mitochondrial oxidative phosphorylation, located at complex I, revealing a temporal delay in females with respect to males associated with a parallel increase in selected markers of protein oxidative damage. Further, females exhibit a fatty acid profile with higher levels of docosahexaenoic acid at 30 days. Mechanistic studies showed that hSOD1-G93A overexpression in N2A cells reduced complex I function, a defect prevented by 17β-estradiol pretreatment. In conclusion, ALS-associated SOD1 mutation leads to delayed mitochondrial dysfunction in female mice in comparison with males, in part attributable to the higher oestrogen levels of the former. This study is important in the effort to further understanding of whether different degrees of spinal cord mitochondrial dysfunction could be disease modifiers in ALS.

**Electronic supplementary material:**

The online version of this article (doi:10.1186/s40478-015-0271-6) contains supplementary material, which is available to authorized users.

## Introduction

Amyotrophic lateral sclerosis (ALS, OMIM #105400), the motor neuron disorder with the highest level of occurrence in adult humans, is characterized by a progressive loss of upper and lower motor neurons that leads to muscular atrophy, paralysis, and death after an average disease duration of 3 years [1]. The most common form of ALS is sporadic, having no apparent inheritability, whereas the dominantly inherited familial ALS accounts for only about 5–10 % of all ALS patients [2, 3]. Among the genetic causes of ALS, 15–20 % of familial ALS cases and about 5 % of sporadic ALS cases are associated with multiple mutations in the gene for Cu,Zn-superoxide dismutase (SOD1), which imparts an increase in a novel toxic function to this otherwise superoxide radical scavenging enzyme in normal conditions [4]. Mutated SOD1 forms misfold, aggregate, and accumulate primarily in spinal cord motor neurons and glial cells of ALS patients [5]. Furthermore, transgenic mice overexpressing mutant forms of the human *sod1* gene develop a progressive motor neuron syndrome similar to the human ALS phenotype [6, 7] and they have been extensively used as an experimental model to gain insight into the pathogenesis of ALS. In particular, one of the most studied mutations is the substitution of glycine for alanine in the 93th residue (hSOD1-G93A).

Nonetheless, individual factors could help to explain the great clinical heterogeneity of this disease, with similar mutations leading to markedly clinically different disease forms. Among these factors, gender differences in many neurodegenerative diseases are observed across epidemiologic studies, pathophysiology, and treatments [8, 9]. ALS is no exception; its incidence (male: female ratio 3:1) and prevalence are higher in men than in women, with a predominance of men with younger disease onset. Clinical phenotypes are also different in male and female patients, especially regarding the site of onset of weakness, as well as cognitive impairment [10, 11]. The aforementioned gender differences have been reported both in studies that included all ALS patients (sporadic and familial) and in familial ALS cases studied separately [12]. Lending further support to the greater occurrence of familial ALS in men than in women, data extracted from the ALSoD website (http://alsod.iop.kcl.ac.uk) revealed a 1.5 male/female ratio for most mendelian ALS-related mutant genes, including SOD1. Moreover, it has been reported that gender can account for variations in the course of disease in familial ALS [13].

Most neurodegenerative disorders involve either causally or consequently mitochondrial abnormalities [14–17]. Although the underlying causes of motor neuron degeneration in ALS remain largely unknown, ALS-causing SOD1 mutations lead to mitochondrial dysfunction, among multiple pathogenic pathways also present in sporadic ALS, such as oxidative stress and endoplasmic reticulum stress [18–21]. Mitochondrial dysfunction may also directly provoke cell death by activating the apoptotic cascade, due to misfolded or aggregated SOD1 that results either in aberrant localization and release of proapoptotic factors or binding to apoptotic inhibitors [22].

Studies in different species have reported that mitochondria in females are more differentiated, and as a result, the mitochondria show greater capacity for and efficiency in substrate oxidation than in males. These features of female mitochondria have been described in nervous tissue, among other organs and tissues [23–27]. On the other hand, results of several authors suggest a greater sensitivity of males to mitochondrial dysfunction compared to females, including higher permeability of the outer and inner mitochondrial membranes with increased translocation of apoptosis-inducing factor to the nucleus in neurons from immature rat brains submitted to hypoxia-ischemia [28]. In brain, among other mitochondrial properties more pronounced in males than females are lower mitochondrial electron transport chain complex activities, diminished mitochondrial mass, and lower mitochondrial membrane potential [29, 30].

Taking this background into account, the aim of this work was to study whether gender-related differences in spinal cord mitochondrial function of a mouse model of ALS could contribute to less severe female phenotypes. We hypothesized that differences in the degree of spinal cord mitochondria dysfunction between male and female hSOD1-G93A mice could explain, at least in part, their different clinical features. These data will be helpful to further understanding of whether different degrees of spinal cord mitochondrial dysfunction are related to the earlier onset and faster disease progression in male ALS patients than in their female counterparts. Moreover, understanding the causes of the sex differences in ALS could yield clues concerning the pathophysiology of this devastating disease.

## Materials and methods

### Chemicals

All reagents, unless stated otherwise, were purchased from Sigmα–Aldrich (St. Louis, MO). Cell culture media, sera, antibiotics, media supplements, and Lipofectamine 2000® were obtained from Invitrogen. 17β-estradiol (E2) was dissolved in ethanol at a concentration of 1 mM, further diluted to 40 μM with culture medium, aliquoted, and stored frozen for up to one month at -20 °C. E2 stock solutions were used for a final concentration of 10 nM.

### Animals and phenotype analyses

A colony of the strain B6SJL-Tg (SOD1-G93A)1Gur/J (JAX catalogue stock number 002726; from now on G93A mice) was purchased from The Jackson Laboratories (Bar Harbor, ME), maintained in the B6SJL background, and genotyped as indicated in Additional file 1. Non-transgenic littermates (from now on Control mice) were used as controls. Animals were maintained under a constant 12 h light–dark cycle in individual cages after weaning (day 21) with temperatures around 22 ± 4 °C, and fed standard rodent chow and water ad libitum.

For the phenotype analyses, animals were weighed weekly, and daily for those under stride length analysis. A third-order polynomial curve was fitted to the age-body weight sets of data with Prism 6.0 software (GraphPad, La Jolla, CA) in order to calculate maximum weight and age of attaining maximum weight, and to interpolate age attaining maximum weight minus 10 %. Neurological scoring, paw print analysis, and end-point determination were performed on alternate days starting from day 60; these are further described in the Additional file 1. All experimental procedures were approved by the Ethical Committee for Animal Testing of the Institut de Recerca Biomèdica de Lleida (IRBLleida) and conformed to Directive 2010/63/EU of the European Parliament.

### Spinal cord sample preparation

Animals were sacrificed by cervical dislocation at indicated times after being fasted overnight. Spinal cord lumbar sections were rapidly excised and kept in ice-cold saline to perform respirometry, or frozen in liquid nitrogen and stored at -80 °C for further analysis, respectively. For fatty acid, oxidative markers and western-blot frozen samples were thawed on ice and homogenized at 0 °C in a buffer containing 180 mM KCl, 5 mM 3-[N-morpholino]propanesulfonic (MOPS) acid, 2 mM ethylenediaminetetraacetic acid (EDTA), 1 mM diethylenetriaminepentaacetic acid, 1 μM freshly prepared butylated hydroxyl toluene (BHT), 10 μg/ml aprotinin, and 1 mM phenylmethylsulfonyl fluoride (PMSF), at pH 7.3 using a Potter-Elvehjem motor-driven glass-Teflon homogenizer. After a brief centrifugation (500 x g, 5 min) to pellet cellular debris, protein concentrations were measured in the supernatants using the Bradford assay.

A small set of animals (*n* = 5 per gender, age 90 days) was employed for immunohistochemistry. Briefly, after rapid extraction of spinal cords, these were fixed using paraformaldehyde and cryoprotected using a sucrose 30 % solution in phosphate buffer. After that, spinal cords sections (16 μm) were obtained using a Leica CM3000 cryostat (Leica Microsystems GmbH, Wetzlar, Germany) and immunohistochemistry was performed using anti-p21 (1:500, ref. ab7960, Abcam Co, Cambridge, UK) and SMI-32 (1:500, Stenberger monoclonals; Biolegend, San Diego, CA).

### Cell culture and transfection

Neuro-2A cell line was obtained from ATCC (#CCL-131) and grown in Advanced-Minimum Essential Medium (MEM) without phenol red supplemented with 10 % heat inactivated fetal bovine serum, 2 mM L-glutamine, 20 U/ml penicillin, and 20 μg/ml streptomycin. Cells were kept at 37 °C in a humidified atmosphere with 5 % CO_2_.

After 10 days of pre-treatment with 10nM E2 or vehicle, cells were harvested, counted, and subcultured in 6-well plates (200,000 cells per well). The day after plating, ransfection was performed using Lipofectamine 2000 (Invitrogen, El Prat de Llobregat, Barcelona, Spain) according to the manufacturer’s instructions. Briefly, lipofectamine and selected DNA plasmids were mixed (1 μg of DNA/1 μL of lipofectamine) with Optimen medium (Invitrogen) for 20 min and the resulting mixture was dispensed to the cell cultures (10 μg DNA per well). The plasmids pEGFP-G93-hSOD1 and pEGFP-wt-hSOD1, expressing G93A mutant or non-mutated human SOD1 tagged with enhanced green fluorescent protein (EGFP), respectively, were kindly provided by Dr Josep Esquerda (Lleida, Spain). The pEGFP expression vector was used for mock transfection.

### High resolution respirometry

Fresh spinal cords were rinsed with ice-cold normal saline and cut into slices with a tissue chopper adjusted to a cut width of 300 μm. O_2_ consumption of sets of 5 lumbar spinal cord slices (LSCS) or suspensions of N2A cells (100,000 cells/mL) was measured at 37 °C with high-resolution respirometry, both in *routine* setup (without additional substrates or effectors) and in permeabilized conditions (including substrates and inhibitors of specific respiratory complexes) using an Oxygraph-2 k (Oroboros Instruments, Innsbruck, Austria) with chamber volumes set at 2 mL as detailed in the Additional file 1.

### Western blot analysis

The content of specific mitochondrial respiratory chain complexes was estimated in Neuro-2A cells using Western blot analysis. Equal amounts of protein (10–25 μg) were separated by SDS-PAGE gels. Proteins were transferred using a Mini Trans-Blot Transfer Cell (BioRad) to polyvinylidene difluoride membranes (Immobilon-P, Millipore). Immunodetection was performed using specific antibodies for the 39 kDa (NDUFA9; CI) subunit of complex I (1:1000), 70 kDa subunit (Flavoprotein) of complex II (1:500), 29 kDa (Rieske iron-sulfur protein; CIII) subunit of complex III (1:1000), and COXI subunit of complex IV (1:1000) (ref. A21344, A11142, A21346, and A6403, respectively; Molecular Probes, Eugene, OR).

Expression of mutant and wild type human SOD1 in transfected N2A cells was immunodetected using a monoclonal antibody specific against human SOD1 (Abcam AB52950). Antibodies to porin (1:5000, A31855, Molecular Probes) and β-actin (1:5000, AB20272, Abcam, Cambridge, MA), as a control for total mitochondrial mass or total protein charge, were also used in order to determine the proportion of protein levels to total mitochondrial mass or total protein content. Appropriate peroxidase-coupled secondary anti-rabbit antibodies (1:40000, Pierce, Rockford, IL, USA) or anti-mouse antibodies (1:10000, GE Healthcare, UK) and chemiluminescence horseradish peroxidase substrate (Millipore, Billerica, MA) were used for primary antibody detection. Signal quantification and recording was performed with a Chemi-Doc unit (Bio-Rad Laboratories, Inc.). Protein concentration was determined with the Bradford method. Data were expressed as arbitrary units.

### Oxidation-derived protein damage marker measurements by gas chromatography coupled to mass spectrometry (GC/MS) and fatty acid analyses

Glutamic semialdehyde (GSA), aminoadipic semialdehyde (AASA), Nε- (carboxyethyl)-lysine (CEL), Nε-(carboxymethyl)-lysine (CML), and Nε-(malondialdehyde)-lysine (MDAL) concentrations in total proteins from spinal cord homogenates were measured with GC/MS as previously described [31] using deuterated internal standards for each protein oxidative modification adduct as further described in the Additional file 1.

Fatty acid analysis in tissue samples was performed as previously described [31] by gas chromatography after fatty acid transesterification. Further details are present in the Additional file 1.

### Cytotoxicity assay and ATP content

Viability of N2A cells was assessed with a lactate dehydrogenase (LDH)Cytotoxicity Assay Kit (Promega, Madison, WI) according to manufacturer’s instructions. Briefly, the amount of LDH released into the culture medium is measured using an enzymatic reaction that results in a red formazan product that can be measured spectrophotometrically. Cell viability was evaluated relative to the total LDH from whole cell lysate and the results were expressed as the percentage of viability versus treated with vehicle and/or mock transfected cells.

ATP content in N2A cell homogenates was measured with the ATP Bioluminiscent Assay Kit (Sigma) according to the manufacturer’s instructions. Results are expressed as nanomole ATP per milligram protein.

### Statistical analysis

All statistical analyses were performed using the SPSS software (SPSS Inc., Chicago, IL) or the Prism software (GraphPad Software). Differences between groups were analyzed with the Student’s t tests or ANOVA with Post-Hoc analyses, after normality of variable distribution was ensured by Kolmogorov-Smirnov test. Correlations between variables were evaluated with the Pearson’s statistic. For multivariate analysis of fatty acid composition, the Metaboanalyst platform was used after autoscaling and log transformation of the relative abundances of their composition [32]. The 0.05 level was selected as the point of minimal statistical significance in every comparison.

## Results and discussion

### Disease onset, clinical evolution and survival in G93A mice show gender dimorphism with a less severe phenotype in female mice

**Fig. 1:**
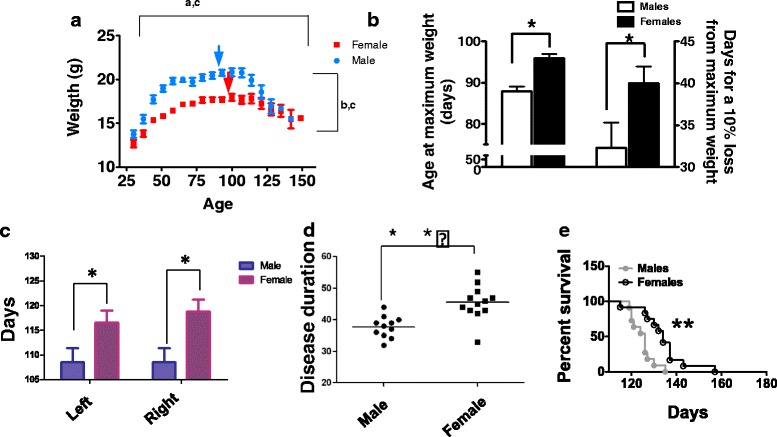
Gender influences weight changes associated to disease onset and progression (**a**, **b**), stride length (**c**), clinical progression (**d**), and survival (**e**) in hSOD-G93A mice. Weight evolution across age in hSOD-G93A mice **a** with *arrows* indicating calculated maximum weight. *Bars* in **b** are means ± SEM of the age at maximum weight or time spent for a 10 % loss from maximum weight, expressed in days. *Bars* in **c** are means ± SEM of the age of clinical onset measured by stride length analysis, expressed in days. *Dots* in **d** shows disease duration among the G93A colony, separated by gender, expressed in days. **e** shows the survival curve. In **a** ^abc^ indicate, significant effects (*p* < 0.01) of age, gender and their interaction, respectively, after 2 way ANOVA. * indicates *p* < 0.05 between male and female mice of the same age with Student’s *t* test. **, *p* < 0.01 after a Kaplan-Meyer analysis

Our G93A mouse colony exhibited a phenotype consistent with the overexpression of the hSOD1-G93A transgene [33]. G93A mice gained weight and reached a higher maximum value for males than for females (21.1 ± 0.2 g vs 18.0 ± 0.1 g, respectively *p* < 0.05) though mice from both genders decreased to a similar body mass when approaching endpoint (Fig. 1a). In comparison, control mice exhibit continuously gender dimorphism in weight (Additional file 2: Figure S1). Onset of clinical symptoms occurred by approximately 70–90 days of age followed by body weight loss, abnormal gait, muscle weakness with decreased grip strength, and impaired coordination. As an estimation of disease onset, we used the interpolated value for age of attaining maximum weight (obtained after fitting a third-order polynomial to the weight evolution curve). Maximum body mass was attained at a significantly younger age in males than in females (87.9 ± 1.2 vs 95.9 ± 1.0 days; *p* < 0.05; Fig. 1a and b). Female G93A mice showed a significant delay in disease progression, as evidenced by slower rate of weight loss (Fig. 1b). As a second estimation of disease progression, stride length was measured with paw print analysis and in this case the onset of clinical weakness was quantified by determining the age at which shortening of the stride length was lower than 40 % for two consecutive measures. These measurements again indicate a slower disease progression in female G93A mice, as this shortening (both for the right and left stride lengths) in G93A females was attained at a significantly older age than in their male counterparts (Fig. 1c). Animals were euthanized when they demonstrated hind limb paralysis or inability to right themselves when placed on their side. Disease duration, estimated as the time between disease onset (10 % loss of their maximum weight) and sacrifice, was significantly longer in females than in males (Fig. 1d). Female G93A mice survived significantly longer than males with survival times of 134.1 ± 2.9 days and 124.9 ± 1.5 days (*p* < 0.01) respectively (Fig. 1e).

Gender appears to modify the course of disease and lifespan in other animal models of ALS with mutations in the hSOD1 gene, with an earlier disease onset in male transgenic rats and mice overexpressing hSOD1-G93A [34, 35], although gender appears to have no consistent effect on survival of ALS patients [36]. To ascertain whether this was due to different expression of hSOD1-G93A protein expression, this parameter was evaluated in spinal cord with western blot analysis, using a monoclonal antibody specific against human SOD1, which has been shown to recognize distinct mutated human SOD1 forms, including hSOD1-G93A [37]. Additional file 2: Figure S2 shows the western blot analysis of transgenic male and female mice at 30 and 60 days, leading to the expression of a protein with an apparent MW of 16 kDa, whereas, as expected, the protein was not detected in control mice. These results provide evidence that while transgenic protein levels, at 30 days, were higher in males, at 60 days this difference was reversed. Thus, at 30 days we cannot rule out different levels of aggregated or aberrant SOD1 activity between male and female transgenic mice as the primary origin of gender differences in survival. However, maximum protein levels of hSOD1-G93A coincide temporally with the late pre-symptomatic stage and the disease onset.

### Oxygen consumption of mouse lumbar spinal cord slices shows early mitochondrial impairment in males linked to complex I dysfunction

**Fig. 2:**
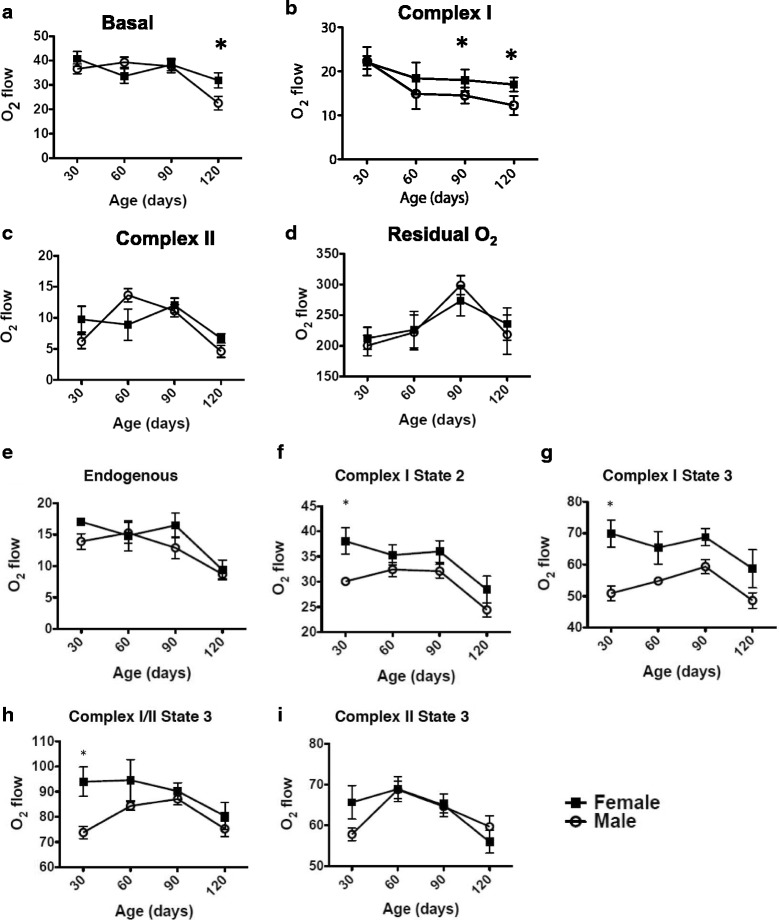
Gender influences O_2_ consumption in lumbar spinal cord slices from hSOD-G93A mice. Graphs shows baseline (**a**), complex I (**b**), complex II (**c**), and residual (**d**) O_2_ consumption in slices from male and female G93A mice. Measurements were taken at 37 °C in Hank’s balanced salt solution containing 10 mM Hepes, pH 7.4, and corrected for instrumental background O_2_ flux. After permeabilization, similar measures were performed for endogenous (**e**), complex I state 2 (**f**), complex I state 3 (**g**), complex I/II state 3 (**h**) and complex II state3 (**i**) O_2_ consumption. Measurements were taken at 37 °C in Mir05 media. Values shown are means ± SEM expressed as pmol O_2_/min · mg protein for 4-6 independent measures. *, *p* < 0.01 with respect to females of the same age with Student’s *t* test

Respirometric analysis of mitochondrial function provides a tool to study changes in mitochondrial respiratory chain function and mitochondrial ATP production in tissue biopsies, cultured cells, and isolated mitochondria [38]. In order to assess whether gender-related differences in spinal cord mitochondrial function could explain longer survival of female animals, we performed high resolution respirometry in lumbar spinal cord slices from male and female mice, both G93A and non-transgenic mice, at several ages. First, using intact (not permeabilized) slices, which resemble in vivo conditions, we found that in G93A mice routine respiration was 41.7 % higher in females at end stage (*p* < 0.05, Fig. 2a), which suggests that mitochondrial function is better spared from disease in female mice, in line with clinical variables.

When comparing the effect of transgene hSOD1-G93A, overexpression only decreased routine O_2_ consumption at the later stages of the disease (control vs. G93A: for males, 37.0 ± 3.8 vs. 22.5 ± 2.7 pmol O_2_/min/mg protein, *P* < 0.01; for females, 40.1 ± 1.5 vs. 31.9 ± 2.8 pmol O_2_/min/mg protein, *p* < 0.01; Additional file 2: Figure S2A and S2B). This may be attributed to the terminal status of the animals and general atrophy of spinal cords at this stage, while no gender differences appeared in control mice (Additional file 2: Figure S3A).

However, considering that this difference was only present at later stages of the disease, we wanted to explore whether any specific respiratory complex was impaired earlier or showed gender related differences. For this reason we studied the sensitivity to complex inhibitors, a validated technique for molecular disection of mitochondrial function, as described in the Materials and methods section. Complex I respiration in G93A females was greater than in males at 60 (20.3 %, *p* = 0.076), 90 (31.4 %, *p* < 0.05), and 120 days (25.6 %, *p* < 0.05) (Fig. 2b). Reinforcing feminine mitochondrial resilience to hSOD1-G93A-linked complex I decay, control-transgenic comparison offered significant differences in complex I function in male mice at all ages studied, whereas transgene only affected complex I activity significantly at later stages of the disease in females (Additional file 2: Figure S2C and S2D). Interestingly, gender-related differences were found in control animals at the earliest ages analyzed. Complex I respiration was 39.6 % higher (*p* < 0.01) in 30-day-old males, whereas in older animals the values were similar in both genders, lacking significant differences (Additional file 2: Figure S3B).

Regarding complex II, no gender differences were found for G93A animals (Fig. 2c) along disease progression. Residual oxygen consumption is due to oxidative side reactions remaining after application of mitochondrial chain complex III inhibitors, such as antimycin A. This has been related to reactive oxygen species (ROS) production. Interestingly, no gender-related differences were found in G93A mice (Fig. 2d). Antimycin A-resistant respiration was higher in control than in G93A of both genders both at 30 days and at 120 days (Additional file 2: Figure S2E and S2F). Interestingly, the opposite results were obtained in residual respiration at 60 days and 90 days, displaying higher rates in male G93A mice at 60 days (Additional file 2: Figure S2E) and in female G93A mice at 90 days (Additional file 2: Figure S2F).

After cell permeabilization for direct evaluation of mitochondrial function no difference was found for endogenous respiration between genders in G93A, supporting the notion that concentration of metabolites of mitochondria do not differ between genders (Fig. 2e). However, complex I-related measures showed higher capacities for females at early stages: state 2 respiration for complex I was higher in females than in males in G93A mice, at a presymptomatic stage (30 days, Fig. 2f). Interestingly, O2 consumption of complex I state 3 was significantly higher in females compared with males all along disease progression (Fig. 2g). Similarly complexI/II state 3 measurements show increased consumption in G93A females in comparison with males (Fig. 2h).

Our results are consistent with previous reports [39] showing that routine (intact tissue, without any inhibitor) respiration only shows evident reduction in G93A animals at the end stage of the disease. Although the routine respiration of intact LSCS was not significantly different in males and female G93A mice (only at endpoint), the higher rotenone resistant respiration in males at 60 days suggests a lower complex I contribution to overall respiration, as calculated by the difference with respect to routine respiration.

Focusing on the G93A animals, females showed greater complex I oxygen consumption than males from 60 days. Nevertheless, this advantage was only significant for 90-day and endpoint animals. Since rotenone-mediated cytotoxicity was proved to be alleviated in female neuron primary cultures [40], this decreased respiration could contribute to the survival demise in males. Studies from [41] showed that, in this mouse model, motor neuron abnormalities begin at 44–60 days. However, cytosol vacuolization and swollen and mega-mitochondria were evident before (30 days, even P7). In line with this we also show early complex I dysfunction in males. This gender dimorphism can be partially explained by previous data [42] showing a protective mechanism of the peroxisome proliferator-activated receptor gamma coactivator 1-alpha (PGC-1α), a master metabolic regulator in mammals. As PGC-1α could enhance the transcriptional activities of sex hormone receptors—e.g., androgen receptor [43] or estrogen receptors [44]—this may result in a feed-back loop, enhancing (in the case of estrogens) survival.

### Oxidative stress markers and fatty acid composition of spinal cord also show gender dimorphism

**Fig. 3:**
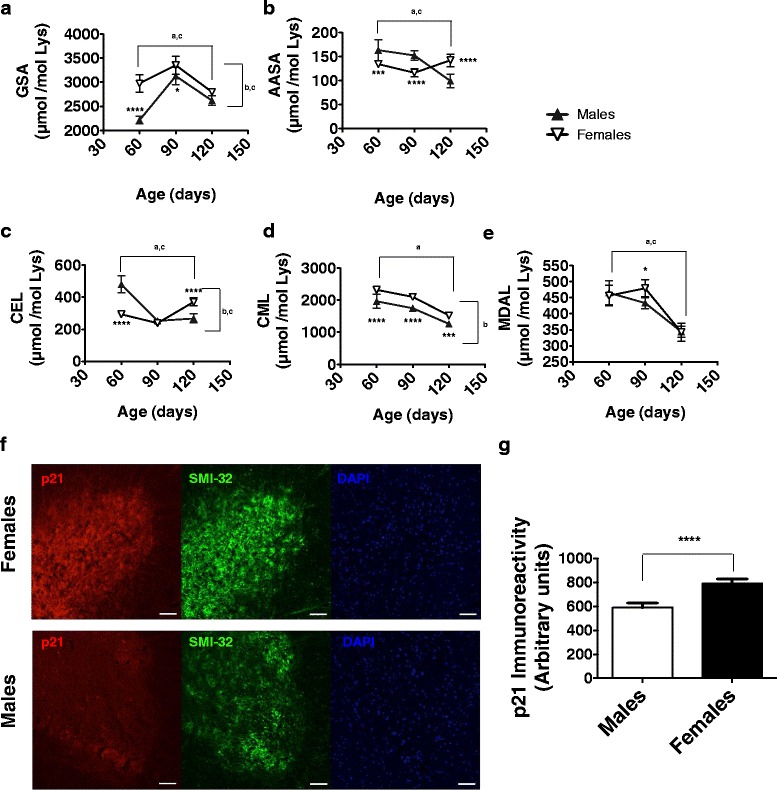
Gender influences protein oxidative modification and p21 expresssion lumbar spinal cords in G93A mice. *Graph* shows values for protein oxidative markers AASA (**a**) and GSA (**b**), protein glycoxidative modifications CEL (**c**) and CML (**d**), and lipid peroxidation derived damage MDAL (**e**). Values shown are means ± SEM expressed as μmol of analyte per mol lysine. **f** Representative immunofluorescence image of lumbar spinal cord slices from G93A mice showing increased p21 levels in females compared with males, as quantified in **g**. ^abc^ indicate, significant effects (*p* < 0.01) of age, gender and their interaction, respectively, after 2 way ANOVA. * shows significant differences for Bonferroni post-hoc (or Student’s *t* test for **g**) analyses (*p < 0.05, **p < 0.01, ***p < 0.001, ****p < 0.0001) between genders at a given age. *Bars* in **e** are 30 μm long

Since complex I dysfunction is linked to ROS production and this is related to fatty acid composition of tissues [45, 46], we measured both structurally defined oxidation products with isotope-dilution GC/MS and fatty acid profiles of spinal cord. In general, both age and gender influenced, in some cases with significative interactions, the concentrations of oxidative modifications in proteins from spinal cord lysates (Fig. 3). Regarding association with longer survival of females, only AASA and CEL showed diminished values in spinal cord at preclinical stages (60 days; Fig. 3b and c), whereas GSA and CML showed increased concentrations in females at these stages (Fig. 3a and d), with MDAL showing no major gender differences. These results contrast with those obtained examining samples of the human disease. In previous analyses, significant increases were reported for ALS samples both in spinal cord and motor cortex [18], especially evident for lipid peroxidation derived markers, such as MDAL. These differences could arise from the effect of overexpressing an antioxidant enzyme (SOD), in the G93A case, which might blunt potential overproduction of ROS linked to ALS. To further confirm differences in ROS production, we also examined expression of p21, a protein regulated by oxidative stress. The results (Fig. 3f and e) are in line with protein oxidative damage, suggesting that at an early clinical stage (90 days) oxidative stress in females is higher than in males. This contrasts with the recently described role of p21 as a potential disease accelerator in astrocytes [47] or an ageing hallmark in neurons [48].

**Fig. 4:**
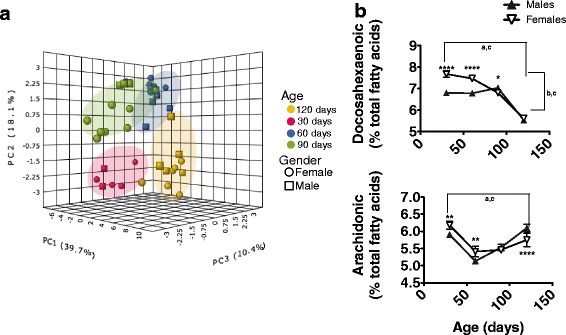
Age and gender are major factors determining lipid composition in lumbar spinal cords in G93A mice. Partial-least square discriminant analyses **a** show that age is the major factor explaining differences in fatty acid composition between G93A samples. Univariate analyses **b** show age- and gender-related changes in the amount of docosahexaenoic and arachidonic fatty acids. ^abc^ indicate significant effects (*p* < 0.01) of age, gender, and their interaction, respectively, after 2 way ANOVA. * shows significant differences for Bonferroni post-hoc analyses (*p < 0.05, **p < 0.01, ****p < 0.0001) between genders at a given age

It is known that fatty acid profile strongly influences membrane peroxidizability. Further, fatty acid composition is associated with changes in survival [49] and mitochondrial function [50]. For these reasons we analysed fatty acid content in G93A mouse spinal cord at different ages. The results (Fig. 4a and Additional file 2: Figure S4) show that in multivariate analyses the major determinant for fatty acid composition of spinal cord in G93A mice was age, with a clear discriminating effect (partial-least square discriminant analyses accuracy 95 %). This was further reinforced by linear general model analyses, in most of which age was the factor with the greatest importance. As an example (Table 1), both for arachidonic and docosahexaenoic acid contents, age appeared as the predictor factor with highest importance. Interestingly, and reinforcing the importance of fatty acid composition in this disease, transgene status also appeared, as the second relevant factor in the amount of these lipids. Finally, and concerning the influence of gender, it strongly influences the levels of structurally and functionally relevant docosahexaenoic acid (Table 1), and its interaction with age and transgen status is relevant in the amount of these fatty acids. Overall, gender, age and transgen status allowed for a model with a high robustness (R^2^ = 0.85).

**Table 1:** Univariant general lineal model linking specific fatty acid amount with gender, G93A-Transgen status and age, as well as its interactions

Variable	22:6 n-3		20:4 n-6	
	F	Sig	F	Sig
Gender	5.372	.023	0.008	.927
G93A Transgen	57.942	.000	25.863	.000
Age	138.614	.000	60.923	.000
Gender-G93A Transgen	12.003	.001	.171	.680
Gender-Age	4.704	.005	4.919	.004
G93A Transgen-Age	7.336	.000	44.616	.000
G93A Transgen-Age-Gender	7.142	.000	1.991	.123

As indicated, the most remarkable change involves the highly peroxidizable docosahexaenoic acid (DHA), which showed a 11.7 % increase in female G93A mouse spinal cord as early as 30 days, with this difference maintained througout the preclinical ages (Fig. 4b, Tables 2, 3, 4 and 5, *p* < 0.01), while it was lost at later stages of the disease. This early increase, based on the neuroprotective role of some derivates of this fatty acid, could contribute to delayed disease phenotypical traits in females. Gender dimorphism in DHA content has not been previously documented in ALS. Observational studies in human children show that females exhibit great benefit from n-3 dietary intake, in terms of cognitive function [51]. This is manifested before sexual maturation, so the role of estrogens and gestagens could be minor. Interestingly, it has been observed that n-3 and progesterone interact in neuroprotection after traumatic brain injury, but information on the interaction of feminine hormones with DHA synthesis or the effects on nervous tissue is scarce. In this sense, epidemiological and basic studies show the potential importace of DHA and other n-3 fatty acids in ALS development [49, 52–54] but to date this is the first report regarding the gender-specific differences in this model.

**Table 2:** Fatty acid composition (% of abundance) spinal cord (30 days)

	Male	Female	*p* value
14:0	2,086 ± 0,005	1,961 ± 0,077	0.2
16:0	23,045 ± 0,182	22,985 ± 0,152	0.8
16:1n-7	1,433 ± 0,043	1,325 ± 0,041	0.2
18:0	17,324 ± 0,026	16,808 ± 0,331	0.2
18:1n-9	35,341 ± 0,041	34,552 ± 0,219	0.03
18:2n-6	1,141 ± 0,063	1,907 ± 0,653	0.3
18:3n-3	0,075 ± 0,001	0,067 ± 0,002	0.1
18:4n-6	0,060 ± 0,001	0,058 ± 0,004	0.7
20:0	0,102 ± 0,003	0,101 ± 0,004	0.8
20:1n-9	1,912 ± 0,058	1,658 ± 0,035	0.1
20:2n-6	0,774 ± 0,033	0,778 ± 0,017	0.9
20:3n-6	0,766 ± 0,048	0,779 ± 0,046	0.9
20:4n-6	5,944 ± 0,040	6,183 ± 0,128	0.1
20:5n-3	0,025 ± 0,001	0,022 ± 0,001	0.2
22:0	0,058 ± 0,003	0,056 ± 0,003	0.8
22:4n-6	2,277 ± 0,069	2,246 ± 0,040	0.8
22:5n-6	0,333 ± 0,013	0,307 ± 0,035	0.5
22:5n-3	0,149 ± 0,013	0,160 ± 0,008	0.6
24:0	0,090 ± 0,001	0,135 ± 0,018	0.1
22:6n-3	6,823 ± 0,090	7,687 ± 0,157	0.01
24:5n-3	0,095 ± 0,007	0,107 ± 0,015	0.5
24:6n-3	0,136 ± 0,006	0,107 ± 0,008	0.1

*p* value reflects statistical significance between hSODG93A Male and Female animals

**Table 3:** Fatty acid composition (% of abundance) spinal cord (60 days)

	Male	Female	*p* value
14:0	1,571 ± 0,050	1,502 ± 0,050	0.3
16:0	20,183 ± 0,106	19,943 ± 0,117	0.1
16:1n-7	1,3876 ± 0,091	1,257 ± 0,063	0.2
18:0	17,072 ± 0,107	17,458 ± 0,187	0.06
18:1n-9	38,651 ± 0,196	37,692 ± 0,230	0.004
18:2n-6	2,059 ± 0,070	1,980 ± 0,032	0.3
18:3n-3	0,082 ± 0,002	0,085 ± 0,005	0.5
18:4n-6	0,205 ± 0,040	0,251 ± 0,007	0.2
20:0	0,4981 ± 0,040	0,454 ± 0,024	0.2
20:1n-9	1,846 ± 0,043	1,802 ± 0,043	0.4
20:2n-6	0,744 ± 0,036	0,764 ± 0,026	0.6
20:3n-6	0,648 ± 0,012	0,666 ± 0,025	0.4
20:4n-6	5,137 ± 0,072	5,411 ± 0,150	0.06
20:5n-3	0,048 ± 0,007	0,275 ± 0,002	0.05
22:0	0,028 ± 0,004	0,019 ± 0,001	0.1
22:4n-6	2,175 ± 0,051	2,338 ± 0,056	0.02
22:5n-6	0,208 ± 0,014	0,239 ± 0,077	0.6
22:5n-3	0,195 ± 0,014	0,190 ± 0,009	0.7
24:0	0,314 ± 0,028	0,295 ± 0,011	0.5
22:6n-3	6,785 ± 0,139	7,475 ± 0,132	0.001
24:5n-3	0,082 ± 0,005	0,058 ± 0,001	0.01
24:6n-3	0,078 ± 0,006	0,082 ± 0,002	0.5

*p* value reflects statistical significance between hSODG93A Male and Female animals

**Table 4:** Fatty acid composition (% of abundance) spinal cord (90 days)

	Male	Female	*p* value
14:0	1,362 ± 0,036	1,395 ± 0,058	0.5
16:0	19,191 ± 0,133	18,902 ± 0,249	0.2
16:1n-7	1,219 ± 0,055	1,317 ± 0,034	0.1
18:0	17,866 ± 0,127	17,735 ± 0,187	0.5
18:1n-9	38,326 ± 0,258	39,209 ± 0,349	0.03
18:2n-6	2,239 ± 0,056	1,903 ± 0,082	0.003
18:3n-3	0,128 ± 0,021	0,111 ± 0,005	0.3
18:4n-6	0,134 ± 0,021	0,102 ± 0,011	0.1
20:0	0,418 ± 0,017	0,478 ± 0,026	0.03
20:1n-9	1,888 ± 0,037	1,782 ± 0,015	0.001
20:2n-6	0,694 ± 0,014	0,605 ± 0,010	0.001
20:3n-6	0,598 ± 0,011	0,533 ± 0,006	0.001
20:4n-6	5,538 ± 0,112	5,466 ± 0,080	0.5
20:5n-3	0,038 ± 0,002	0,051 ± 0,006	0.07
22:0	0,017 ± 0,001	0,038 ± 0,006	0.004
22:4n-6	2,357 ± 0,086	2,353 ± 0,042	0.9
22:5n-6	0,296 ± 0,015	0,385 ± 0,059	0.2
22:5n-3	0,202 ± 0,004	0,259 ± 0,011	0.001
24:0	0,295 ± 0,018	0,409 ± 0,021	0.002
22:6n-3	7,039 ± 0,107	6,808 ± 0,067	0.05
24:5n-3	0,060 ± 0,001	0,084 ± 0,008	0.01
24:6n-3	0,090 ± 0,005	0,124 ± 0,012	0.01

*p* value reflects statistical significance between hSODG93A Male and Female animals

**Table 5:** Fatty acid composition (% of abundance) spinal cord (120 days)

	Male	Female	*p* value
14:0	1,128 ± 0,243	1,461 ± 0,107	0.1
16:0	19,346 ± 0,314	19,983 ± 0,380	0.07
16:1n-7	1,193 ± 0,178	1,338 ± 0,193	0.1
18:0	17,274 ± 0,243	17,452 ± 0,206	0.3
18:1n-9	40,311 ± 0,559	39,427 ± 0,454	0.1
18:2n-6	2,076 ± 0,071	1,968 ± 0,120	0.5
18:3n-3	0,149 ± 0,005	0,146 ± 0,006	0.7
18:4n-6	0,214 ± 0,010	0,207 ± 0,014	0.4
20:0	0,429 ± 0,020	0,497 ± 0,038	0.1
20:1n-9	1,644 ± 0,018	1,675 ± 0,064	0.8
20:2n-6	0,502 ± 0,022	0,478 ± 0,017	0.1
20:3n-6	0,619 ± 0,029	0,591 ± 0,023	0.2
20:4n-6	6,101 ± 0,112	5,758 ± 0,171	0.06
20:5n-3	0,078 ± 0,016	0,090 ± 0,012	0.6
22:0	0,069 ± 0,005	0,095 ± 0,007	0.03
22:4n-6	2,138 ± 0,068	1,902 ± 0,059	0.008
22:5n-6	0,193 ± 0,017	0,226 ± 0,033	0.3
22:5n-3	0,244 ± 0,007	0,247 ± 0,011	0.9
24:0	0,584 ± 0,023	0,659 ± 0,039	0.1
22:6n-3	5,533 ± 0,055	5,612 ± 0,100	0.7
24:5n-3	0,061 ± 0,008	0,062 ± 0,004	0.9
24:6n-3	0,106 ± 0,012	0,116 ± 0,014	0.6

*p* value reflects statistical significance between hSODG93A Male and Female animals

Concerning the potential mechanisms connecting enhanced DHA amount and female resilience to ALS development, it should be recalled that DHA is a precursor of endogenous modulators of neuroinflammation [55]. One of these modulators, resolvin D1 shows a relevant role as inhibitor of inflammatory cytokine secretion in the same model we have examined [56].

### Overexpression of HSOD1-G93A in Neuro 2A cells leads to a loss in complex I function which can be prevented by estradiol

**Fig. 5:**
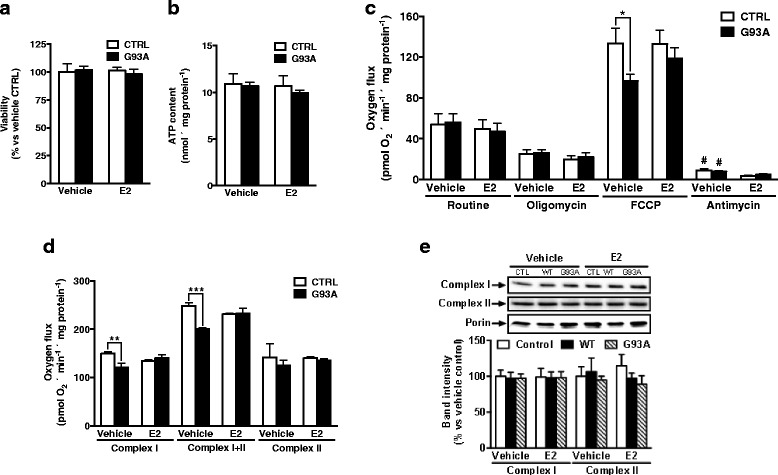
Overexpression of HSOD1-G93A in N2A cells reproduces complex I function loss, preventable by estradiol treatment. Neither cell viability **a** nor ATP content **b** was altered as a consequence of transfection or estradiol treatment. **c** O_2_ flux measurements in intact N2A cells pretreated with E2 and transfected with pEGFP-HSOD1-G93A and pEGFP-hSOD-wt showed a reduction in the maximum oxygen capacity in G93A transfected cells which was abrogated by estradiol treatment. **d** O_2_ flux measurements in above-mentioned permeabilized cells showed a complex I impairment for cells transfected with G93A with respect to control cells which was abolished by the estradiol treatment. In the same experimental regime, western blot analysis **e** for mitochondrial complex protein expression (complex I and complex II) confirmed an unaltered representative peptide content across different treatment and transfects. *Bars* in **a** represent % of survival ± SEM respect to control cells for 4-6 independent experiments. *Bars* in **b** represented ATP mean values ± SEM expressed as nmol/mg protein for 4-6 independent experiments. O_2_ consumption in **b** was measured at 37 °C in culture medium containing 10 mM Hepes, pH 7.4, and corrected for instrumental background O_2_ flux. In **d** culture medium was MIR05. Values were normalized for actual protein content in the respirometer chambers, expressed as pmol O2/min · mg protein. *, *P* < 0.05; **P < 0.01; ***P < 0.001 between control cells and G93A cells for 4-6 independent experiments. *Bars* in **e** represent means ± SEM densitometric analysis of the corresponding complexes relativized for actual porin content in each lane for 4-6 independent experiments

Higher estrogen level in females could explain the gender-related differences, since these hormones have mitochondrial effects [57, 58]. To evaluate whether these could explain gender differences in mitochondrial function in G93A mice, we studied the effects of estradiol preincubation (10 days) in N2A cells expressing either wild type hSOD1 or G93A-mutated hSOD1 (both containing EGFP to control transfection, which achieved 80–90 % efficiency (Additional file 2: Figure S5). There was no significant difference in the cell viability between these treatments (Fig. 5a). Cellular ATP content showed similar results, without major effects of G93A mutation or estradiol treatment (Fig. 5b). Mitochondrial function was evaluated as above. In line with the lack of differences in ATP concentrations, O_2_ consumption, both in standard culture medium (without additional substrates, inhibitors, or uncoupling agents) and in non-phosphorylating conditions (after inhibition of mitochondrial chain complex V F0-ATP synthase with oligomycin), was not different among treatments (Fig. 5c). This could be explained by the Warburg effect [59], in which tumoral cells in usual culture media (having enough glucose) could favour glycolytic procedures over mitochondrial respiration for ATP production. To establish whether mitochondrial functional reserves were impaired, we measured maximum respiratory capacity (obtained after uncoupling). In line with in vivo G93A effect in mitochondrial function, the transfection of G93A-hSOD significantly reduced (*p* < 0.05) maximal respiratory capacity (Fig. 5c). Interestingly, estradiol pretreatment abbrogated these differences, suggesting that estrogens could explain the preserved mitochondrial function present in female transgenic mice. As this respiration was inhibited by antimycin A to an equal extent in all the situations, these results point to a complex I malfunction. To confirm this, we measured O_2_ consumption after cell permeabilization (in the presence of mitochondrial substrates and inhibitors). In line with the above results, neither endogenous respiration (before addition ofs ubstrates, data not shown) nor complex I state 2 respiration stimulated by glutamate/malate were significantly different in the examined conditions (data not shown). However, after the addition of ADP, in order to achieve complex I state 3 respiration, O2 uptake in G93A-hSOD transfected cells was lower than in wild type-hSOD or mock EGFP transfected cells (Fig. 5d, complex I). This respiration difference was enhanced after the addition of succinate (Fig. 5d, complex I + II), but disappeared when rotenone was injected in the measurement chambers (Fig. 5d, complex II), reinforcing the role of complex I dysfunction. Importantly, estradiol preincubation prevented the hSOD1-G93A induced differences. In order to assess the origin of the reduced complex I function, we estimated the protein expression of complex I by measuring representative subunits (complex I: 39KDa,NDUFA9; complex II: 70KDa, Flavoprotein; complex III: 29 kDa Rieske iron-sulfur protein; complex IV: COXI subunit of complex IV). Figure 5e shows that levels of complex I peptides do not differ among the treatments, suggesting that either the dysfunction is induced by loss of other peptides and/or by dysfunction of complex I without changes in the amount of their components. Levels of the other peptides examined were not different (data not shown). Considering that estrogens do not lead to changes in mitochondrial complex expression, this suggest that there is a greater efficiency in terms of ATP production in females. This could enhance their homeostatic capacities, therefore supporting an enhanced response against bioenergetic failure in spinal cord of G93A mice and, possibly, ALS patients, by changing the expression of other mitochondrial proteins. In line with this, researchers have focused on gender relevance in the mitochondrial permeability transition pore formation in the hSOD1-G93A mouse model [60]. Hence, they demonstrated that ablation of cyclophilin D (a protein structurally linked to the mitochondrial permeability membrane transition pore) completely abolished the phenotypic advantage of female hSOD-G93A, with no major effect found in males. In fact, they showed that 17β-estradiol protected hSOD1-G93A expressing cortical neurons and spinal cord motor neurons against glutamate toxicity in brain mitochondria, but the protection was lost in neurons lacking cyclophilin D. All in all, our results suggest that mitochondrial complex activities could be relevant disease modifiers in ALS. Confirming this, very recent data [61] show that even in peripheral blood cells, ALS is associated with decreased complex I activity.

As limitations of this work we acknowledge that measurements performed in total spinal cord, either fatty acid composition, oxidative damage or mitochondrial function, do not allow to specify the cell involved. Thus, we do not know whether the in vivo effect of gender is located in neurons, glia or both. Also, important differences in oxidative metabolism in skeletal muscle between males and females cannot be neglected [24], and it may be very relevant in ALS development. Further, a recent metaanalysis review indicates that some of the gender related differences in survival present in this model may be related to the genetic background [62]. This indicates that the relationship between gender, disease development and mitochondrial function is complex. Furthermore, mitochondrial vacuolisation and degeneration is a dominant and early phenomenon in the mouse used in this study, a fact that may be attributed to excessive SOD1 overexpression. However, the in vitro experiments demonstrating loss of complex I function, without cell death, and the preventive role of estrogens in this phenomenon, point out that even subtle endocrine effects on mitochondrial function could be relevant at long term. The mechanisms behind the gender and background sparing effects on the pathogenic burden of overexpression of mutant SOD1 merit further exploration, but our data show that this should comprise evaluation of mitochondrial function and fatty acid composition.

Even accounthing these limitations, we have described an early mitochondrial defect during the presymptomatic stage associated with the expression of G93A-mutated human SOD1 that appears at a younger age in male mice than in females, correlating with the delay in the clinical features of ALS-like phenotype.

## Conclusions

Our data support a role for dysfunctional mitochondria in ALS pathogenesis and, in particular, the gender bias towards males observed in ALS. This is added to changes in fatty acid composition and oxidative stress markers. These factors could be disease modifiers, affecting the phenotypic differences among ALS patients. Furhter, mitochondrial targets such as complex I may represent a viable focus for novel treatments of a range of disorders affecting motor systems. The presented data, finally, underlines the importance of further studies directed at establishing the role of sex hormones and related molecules in an ALS cure.

## Additional files

Additional file 1:
**Supplemental materials and methods.**(DOCX 149 kb) Additional file 2:
**Supplemental results.**(PDF 5179 kb)

## Abbreviations

AASA: aminoadipic semialdehyde
ALS: amyotrophic lateral sclerosis
BHT: butylated hydroxyl toluene
CEL: Nε- (carboxyethyl)-lysine
CML: Nε- (carboxymethyl)-lysine
DHA: docosahexaenoic acid
E2: 17β-estradiol
EDTA: ethylenediaminetetraacetic acid
EGFP: enhanced green fluorescent protein
GC/MS: gas-chromatography coupled to mass spectrometry
GSA: glutamic semialdehyde
hSOD-G93A: Cu,Zn superoxide dismutase with the G93A mutation
LDH: lactate dehydrogenase
LSCS: lumbar spinal cord slices
MDAL: Nε-(malondialdehyde)-lysine
MOPS: 3-[N-morpholino]propanesulfonic acid
N2A: Neuro2A
PGC-1α: per
PMSF: phenylmethylsulfonyl fluoride
ROS: reactive oxygen species
SOD1: Cu,Zn-superoxide dismutase
