# Supplementary material for: Early and gender-specific differences in spinal cord mitochondrial function and oxidative stress markers in a mouse model of ALS
Source: Acta Neuropathol Commun. 2016 Jan 13;4:3. doi: 10.1186/s40478-015-0271-6 (PMC4711180; doi:10.1186/s40478-015-0271-6)
Supplement: Additional file 1: — Supplemental materials and methods.(DOCX 149 kb) [file 40478_2015_271_MOESM1_ESM.docx]

**Supplemental methods**

- - 1. Genotyping

Mice were identified by PCR with tail genomic DNA as templates. The REDExtract-N-Amp Tissue PCR Kit (XNAT; Sigma-Aldrich, St. Louis, MO) was used to extract genomic DNA from mouse tails and amplify targets of interest by PCR according to manufacturer’s instructions. Four primers (a pair for wild type forward: 5´-CTA GGC CAC AGA ATT GAA AGA TCT-3´ and reverse: 5´-GTA GGT GGA AAT TCT AGC ATC ATC-3´ and for G93A forward: 5´-CAT CAG CCC TAA TCC ATC TGA-3´ and reverse: 5´-CGC GAC TAA CAA TCA AAG TGA-3´) were used to amplify selected bands from the inserted targeting vector. Briefly, the DNA is released from the tail tissue by incubating the sample with a mixture of the Extraction Solution and the Tissue Preparation Solution at room temperature for 10 minutes. After adding Neutralization Solution B, the extract is ready for PCR. A 4 μL aliquot of the neutralized extract is then combined with 10 μL of 2x REDExtract-N-Amp PCR Reaction Mix (buffer, salts, dNTPs, Taq polymerase, JumpStart Taq antibody and REDTaq® dye) and appropriate volumes of each primer and water (PCR grade; Sigma-Aldrich, St. Louis, MO). PCR conditions were: after 1 cycle of 3 minutes at 94°C, 35 cycles of 30 seconds at 94°C, 30 seconds at 66°C, and 1 minute at 72°C, with a final extension at 72°C for 10 min, using an Applied Biosystems 2740 v.2.08 (Carlsbad, CA) thermal cycler. As a control of effective PCR amplification, housekeeping gene interleukin 5 was used. The resulting PCR products were stored at 4˚C until direct loading onto an agarose gel. Products were electrophoresed for 30 minutes under constant voltage (100V) and imaged under a UV light (302 or 365 nm) lamp from Alpha Innotech (Santa Clara, CA) and analysed with Alpha Digidoc RT2 software (Santa Clara, CA).

- - 1. Neurological scoring

Neurological scoring was performed everyday for both hind legs for each mouse from 60 days of age. The neurological score employed a scale of 0 to 4 which was a slight variation of the scale developed by observation at Amyotrophic Lateral Sclerosis Therapy Development Institute (ALSTDI). Criteria used to assign each score level were as follows: score 0, full extension of hind legs away from lateral midline when mouse is suspended by its tail, and mouse can hold this for 2 seconds, suspended 2–3 times; score 1, collapse or partial collapse of leg extension towards lateral midline or trembling of hind legs during tail suspension; score 2, toes curl at least twice during walking a distance of 40cm, or when any part of the foot is dragged along the cage bottom or table (if one hind leg is scored as 2, food pellets are left on bedding and a long sipper tube is placed on the water bottle); score 3, rigid paralysis or minimal joint movement, foot not being used for forward motion; and, score 4, mouse can not right itself within 30 seconds from either side. When mice achieved a score of 4 for two alternate days, they were euthanized.

- - 1. Paw print analysis

Mice were trained to walk freely along a U-shaped plastic corridor (5 cm wide, 70 cm long) three times per week before data collection (average age 70 days). Once trained to perform stride length measurements, hind limbs were stained with non-toxic children paint and their tracks were imprinted on paper lining the floor of the corridor. Only continuous runs were selected among recorded paw prints and a minimum of 5-7 strides were measured per animal per day (measures were taken three times per week). Since animals tended to run when released and walk slowly at the end of the corridor, those sections of the paw print were discarded for the stride length measurements. Three stride length measurements were taken manually as described previously [1]: front and back stride, and front-to-back distance. Fifty total measures were taken for each measurement. Front stride and back stride were collected as a straight line from paw print to the following paw print. Front-to-back distance was collected as a straight line from back paw print to corresponding front paw print. Correspondence was based on closest front footprint. The distance was recorded as length of line from paw to the stride line opposite the paw print. Since only number of the randomized animal and date identified the recorded tracks, measurement of stride length was determined in a blinded manner. The onset of clinical weakness was quantified by determining the age at which shortening of the stride length was lower than 40% for two consecutive measures.

*Respirometry analysis*

Spinal cord permeabilization was a modification of a previously published method[2]. About 12-15 slices of the lumbar region were collected and transferred quickly into individual wells of a 6-well tissue culture plate with 2 ml of ice-cold permeabilization medium (in mM: 7.23 potassium ethylene glycol-bis(b-aminoethyl ether)*N*,*N*,*N’*,*N’*-tetraacetate (K_2_ EGTA), 2.77 CaK_2_ EGTA, 60 *N*,*N*-bis[2-hydroxyethyl]- 2-aminoethanesulfonic acid (BES), 5.69 MgATP, 20 taurine, 3 K_2_HPO4 , 0.5 dithiothreitol and 81 potassium methanesulfonate, pH 7.1 at 25ºC), rinsed and immediately transferred again into another well with the same medium containing 20 μl of saponin stock solution (5 mg/ml; final concentration 50 μg/ml). Lumbar spinal cord slices (LSCS) were then shaken by gentle agitation in the cold room (on ice) for 30 min. Afterwards, all samples were quickly transferred from the saponin permeabilization medium into 2 ml of respiration medium (in mM: 7.23 K_2_ EGTA, 2.77 CaK_2_ EGTA, 100 potassium salt of 2-(N-morpholino)ethanesulfonic acid (K-MES), 1.38 MgCl_2_ , 20 taurine, 3 K_2_HPO4 , 0.5 dithiothreitol, 20 imidazole and 5 mg/ml bovine serum albumine (BSA), pH 7.1 at 25ºC), and shaken by gentle agitation for 10 min in the cold room (on ice) before performing respirometry.

In order to avoid tissue disaggregation, LSCSC required setting a slow bar stirring speed (150 rpm), but not too low to compromise homogeneity of substrate and oxygen concentrations in the measuring chambers, and, therefore, signal stability. Stirring speed for suspensions of N2A cells was set to 400 rpm after a trypan blue exclusion assay showed cell viability was > 95% (data not shown) after 1 h of agitation. DatLab software (Oroboros Instruments, Innsbruck, Austria) was used for data acquisition (2 s time intervals) and analysis, which includes calculation of the time derivative of oxygen concentration and correction for instrumental background oxygen flux[3].

Respiration of LSCS and N2A cells was analysed following two standardized protocols. The experimental regime for intact LSCS and N2A cells started with routine respiration, which we defined as the oxygen flux in Hank’s balanced solution and cell culture medium, respectively, without additional substrates or effectors. After observing steady-state respiratory flux in the time interval between 5 and 10 min after closing the chamber, respiration was inhibited by sequential addition of rotenone at 0.5 μM (to test for the effect of inhibiting complex I activity) and antimycin A at 2.5 μM (inhibiting complex III). This titration method was completed within 30 min. In order to avoid oxygen limitations, all the experiments were performed above 50% oxygen saturation. Oxygen consumption was normalized for actual protein content and citrate synthase activity in the respirometer chambers.

The second experimental regime required tissue or cell permeabilization before placing the sample in the measurement chamber. Initially, we measured endogenous respiration in the absence of additional substrates. For evaluation of relative contributions of mitochondrial complexes to oxygen consumption, several specific mitochondrial inhibitors and substrates were added sequentially as routinely performed in our laboratory and calculated as steady-state respiratory flux in the time interval between 5 and 10 min after their addition. First, we added glutamate (10mM) and malate (5mM) to increase NADH levels in order to measure the complex I non-phosphorylative activity, or state 2. 10 mM ADP was added to quantify the complex I-dependent phosphorylative activity, or state 3. Immediately afterwards, we added succinate (10mM), which is the substrate for complex II. At this point, the level of oxygen consumption corresponded to complex I- and II-dependent phosphorylative activity. The addition of 0.5 μM rotenone inhibits complex I; therefore, oxygen consumption measured after the addition of rotenone only reflects complex II-dependent phosphorylative activity (in the absence of electron back flux to complex I). Then, complex III activity was inhibited with antimycin A (2.5 μM), and finally complex IV maximal activity was measured after addition of the non-physiological substrate *N,N,N’,N’*-tetramethyl-*p*-phenylenediamine (TMPD) at 0.5 mM, ascorbate (2mM) and cytochrome c (10 μM). Maximal oxygen consumption rates of spinal cords were measured after addition of carbonyl cyanide-p-trifluoromethoxyphenylhydrazone (FCCP). In order to avoid oxygen limitations, all the experiments were performed above 50% oxygen saturation. Oxygen consumption was normalized for actual protein content in the respirometer chambers.

*Analyses of protein oxidative modifications*

Samples containing 0.75-1 mg of protein were delipidated using chloroform:methanol (2:1 v/v), and proteins were precipitated by adding 10% trichloroacetic acid (final concentration) and subsequent centrifugation. Protein samples were reduced overnight with 500 mM NaBH4 (final concentration) in 0.2M borate buffer, pH 9.2, containing 1 drop of hexanol as an anti-foam reagent. Proteins were then reprecipitated by adding 1ml of 20% trichloroacetic acid and subsequent centrifugation. The following isotopically labelled internal standards were then added: [2H8]Lysine (d8-Lys; CDN Isotopes); [2H4]CML (d4-CML), [2H4]CEL (d4-CEL), and [2H8]MDAL (d8-MDAL), prepared as described (8,26); [2H5] 5-hydroxy-2-aminovaleric acid (for GSA quantization) and [2H4]6-hydroxy-2-aminocaproic acid (for AASA quantization) prepared as described in [4]. The samples were hydrolysed at 155ºC for 30 min in 1ml of 6N HCl, and then dried in vacuo. The N,O-trifluoroacetyl methyl ester derivatives of the protein hydrolysate were prepared as previously described[4]. GC/MS analyses were carried out on a Hewlett-Packard model 6890 gas chromatograph equipped with a 30m HP-5MS capillary column (30m x 0.25mm x 0.25 μm) coupled to a Hewlett-Packard model 5973A mass selective detector (Agilent, Barcelona, Spain). The injection port was maintained at 275ºC; the temperature program was 5 min at 110ºC, then 2ºC/min to 150ºC, then 5ºC/min to 240ºC, then 25ºC/min to 300ºC, and finally a hold at 300ºC for 5 min. Quantification was performed by external standardisation using standard curves constructed from mixtures of deuterated and non-deuterated standards. Analytes were detected by selected ion-monitoring GC/MS. The ions used were: lysine and d8-lysine, m/z 180 and 187, respectively; 5-hydroxy-2-aminovaleric acid and d5-5-hydroxy-2-aminovaleric acid (stable derivatives of GSA), m/z 280 and 285, respectively; 6-hydroxy-2-aminocaproic acid and d4-6-hydroxy-2-aminocaproic acid (stable derivatives of AASA), m/z 294 and 298, respectively; CML and d4-CML , m/z 392 and 396, respectively; CEL and d4-CEL, m/z 379 and 383, respectively; and MDAL and d8-MDAL, m/z 474 and 482, respectively. The amounts of products were expressed as the ratio μmol GSA, AASA, CML, CEL or MDAL per mol lysine.

*Fatty acid analysis*

Total lipids from homogenates were extracted with chloroform/methanol (2:1, v/v) (3 times) in the presence of 0.01% butylated hydroxytoluene. The chloroform phase was evaporated under nitrogen, and the fatty acids were transesterified by incubation in 2 ml of 5% methanolic HCl for 90 min at 75 °C. The resulting fatty acid methyl esters were extracted by adding 2 ml of n-pentane and 1 ml of saturated NaCl solution. The n-pentane phase was separated, evaporated under nitrogen, and redissolved in 80 μl of carbon disulfide, and then 2 μl was used for GC analysis. Separation was performed with a DBWAX capillary column (30 m×0.25 mm×0.20 μm) in a GC System 7890A with a Series Injector 7683B and a FID detector (Agilent Technologies, Barcelona, Spain). The injection port was maintained at 220°C and the detector at 250°C; the temperature program was 2 min at 100°C, then 10°C/min to 200°C, then 5°C/min to 240°C, and finally a hold at 240°C for 10 min. Identification of fatty acid methyl esters was made by comparison with authentic standards (Larodan Fine Chemicals, Malmö, Sweden). Results are expressed as mol%.

**Legends to supplemental figures**

Supplemental figure 1. Weight changes in control littermates. Weight evolution across the study for controls. Dots show means ±SEM values in grams n=12 *, p<0.05 between male and age matched females after Student’s t test.

Supplemental figure 2. Time course of G93A-hSOD1 protein expression in G93A mice spinal cord. Western-blot analysis of transgenic male and female mice at 30 and 60 days leads to the expression of a protein with an apparent MW of 16 kDa, whereas, as expected, the protein was not detected in control mice.

Supplemental figure 3. O_2_ consumption of sets of 5 intact LSCS from male and female G93A and control mice measured at 30, 60, 90, and 120 (endpoint) days of age. Experimental procedures allow measurement of baseline (A-B), complex I (C-D), and residual (E-F) O_2_ consumption differences between G93A and controls, depicted by sex. Measurements were taken at 37 °C in Hank’s balanced salt solution containing 10mM Hepes, pH 7.4, and corrected for instrumental background O_2_ flux. Values were normalized for actual protein content in the respirometer chambers. Means ± SEM, expressed as pmol O_2_/s·mg protein. *, p<0.05, between male and female mice of the same age by Student’s t test.

Supplemental figure 4. O_2_ consumption of sets of 5 LSCS from male and female control mice measured at 30, 60, 90, and 120 (endpoint) days of age. In the upper panel, intact measures showed baseline (A), complex I (B) and complex II (C) oxygen consumption differences between males and age-matched females in the control colony. Measurements were taken at 37 °C in Hank’s balanced salt solution containing 10 mM Hepes, pH 7.4, and corrected for instrumental background oxygen flux. Values shown are means ± SEM expressed as pmol O_2_/min·mg protein for 4-6 independent measures. *, p < 0.01 respect to females of the same age by Student’s t test.

Supplemental figure 5. Heat map showing relative fatty acid abundance across different ages and genders in G93A colony.

Supplemental figure 6. Phase contrast (A, B) and fluorescence (C, D) microscopy images of the N2A culture after pEGFP-hSOD-wt (A) and (C) or pEGFP-G93A-hSOD1 (B) and (D) transfection. Trasfection efficiency reached 80-90%.

**References**

1. Chiu AY, Zhai P, Dal Canto MC, et al. (1995) Age-dependent penetrance of disease in a transgenic mouse model of familial amyotrophic lateral sclerosis. Mol Cell Neurosci 6:349–62. doi: 10.1006/mcne.1995.1027

2. Safiulina D, Kaasik A, Seppet E, et al. (2004) Method for in situ detection of the mitochondrial function in neurons. J Neurosci Methods 137:87–95. doi: 10.1016/j.jneumeth.2004.02.027

3. Gnaiger E (2001) Bioenergetics at low oxygen: dependence of respiration and phosphorylation on oxygen and adenosine diphosphate supply . Respir Physiol 128:277–297.

4. Pamplona R, Dalfó E, Ayala V, et al. (2005) Proteins in human brain cortex are modified by oxidation, glycoxidation, and lipoxidation. Effects of Alzheimer disease and identification of lipoxidation targets. J Biol Chem 280:21522–30. doi: 10.1074/jbc.M502255200
