# Supplementary material for: Early and gender-specific differences in spinal cord mitochondrial function and oxidative stress markers in a mouse model of ALS
Source: Acta Neuropathol Commun. 2016 Jan 13;4:3. doi: 10.1186/s40478-015-0271-6 (PMC4711180; doi:10.1186/s40478-015-0271-6)

Supplemental Figure 1

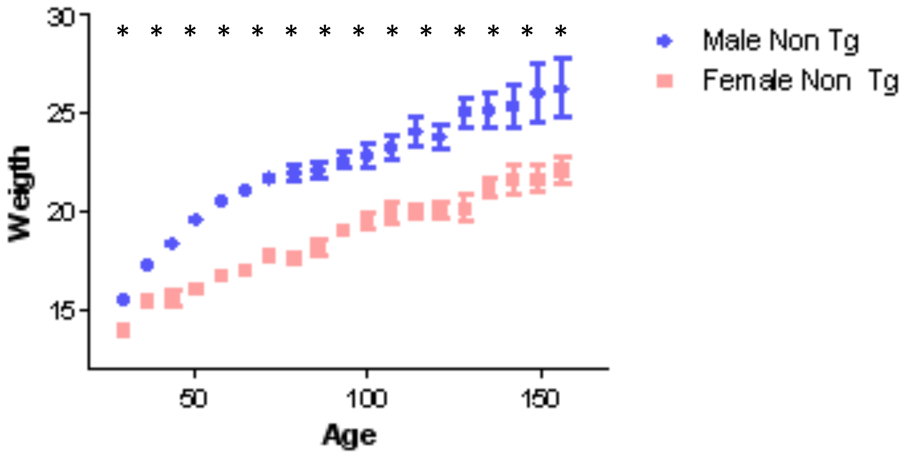

Supplemental Figure 2

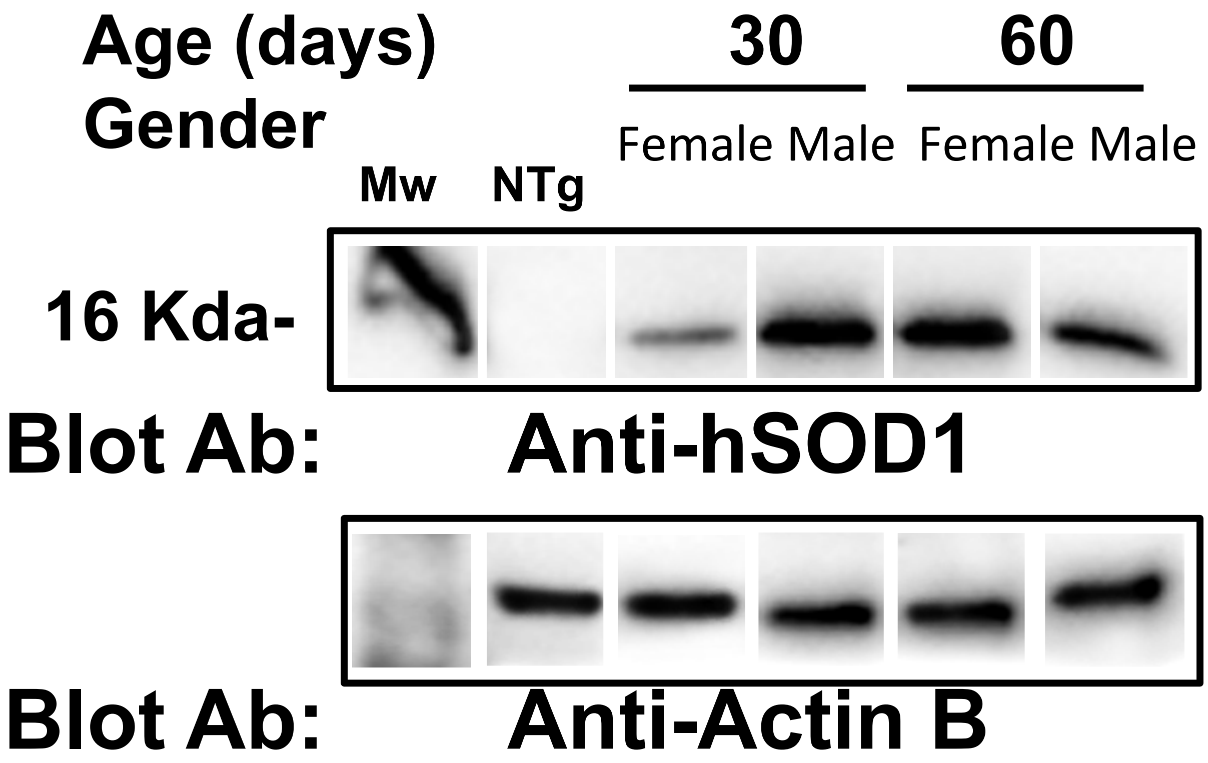

Supplemental figure 3

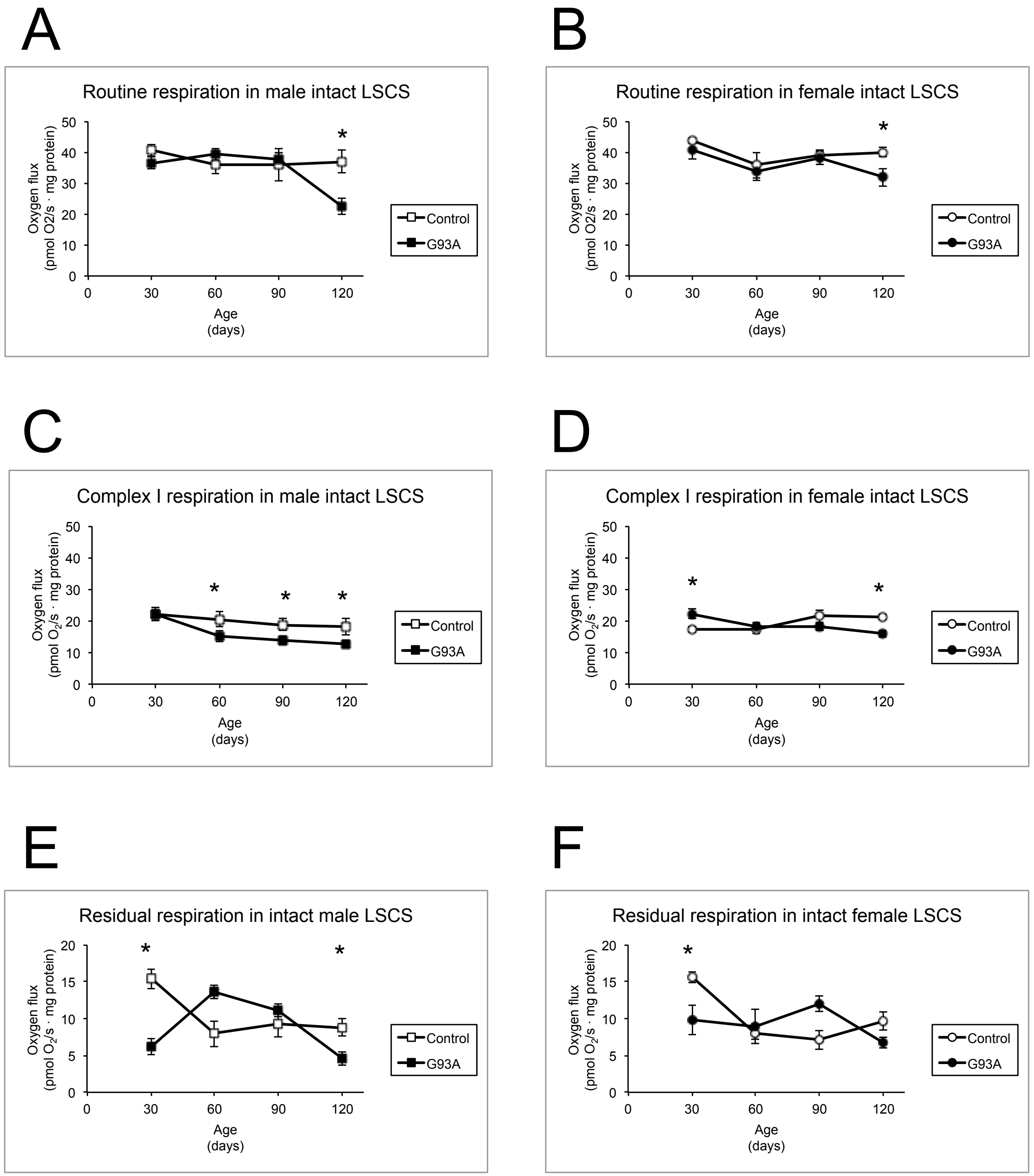

Supplemental figure 4

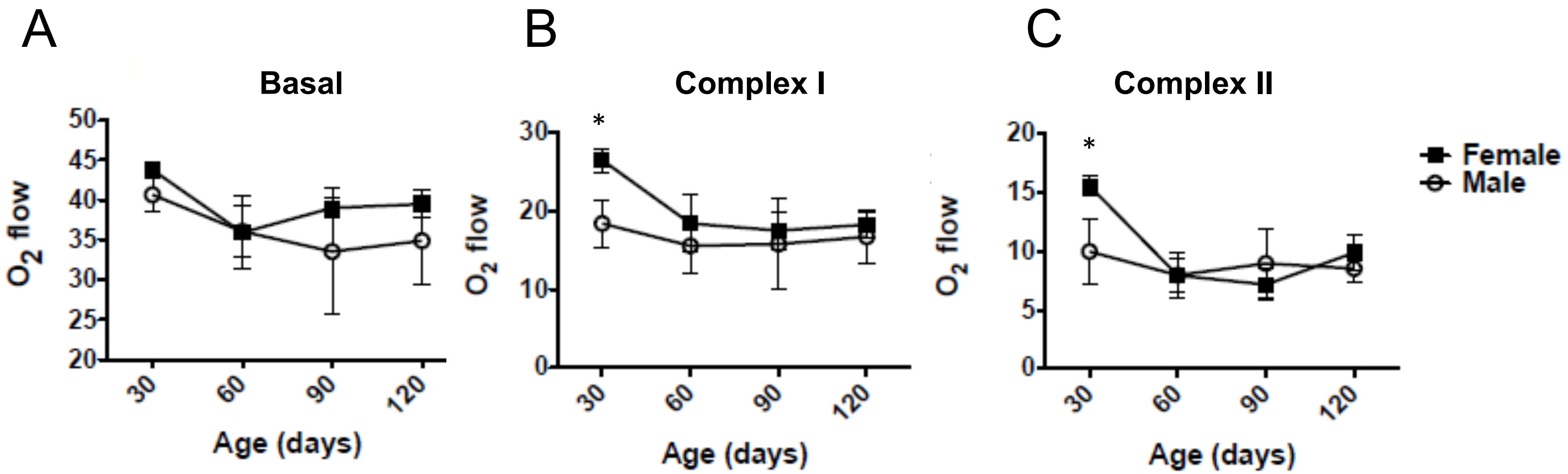

Supplemental figure 5

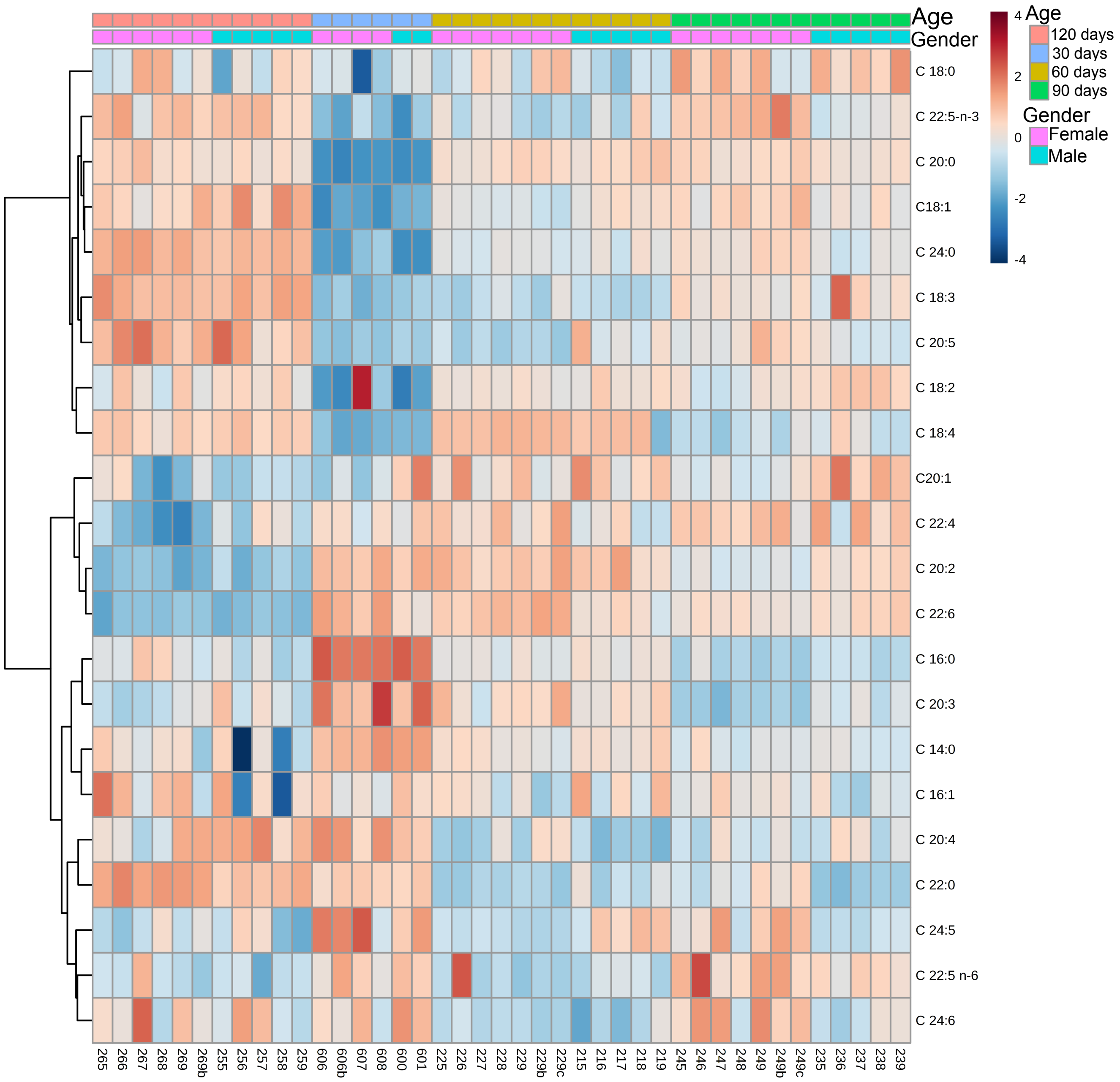

Supplemental figure 6

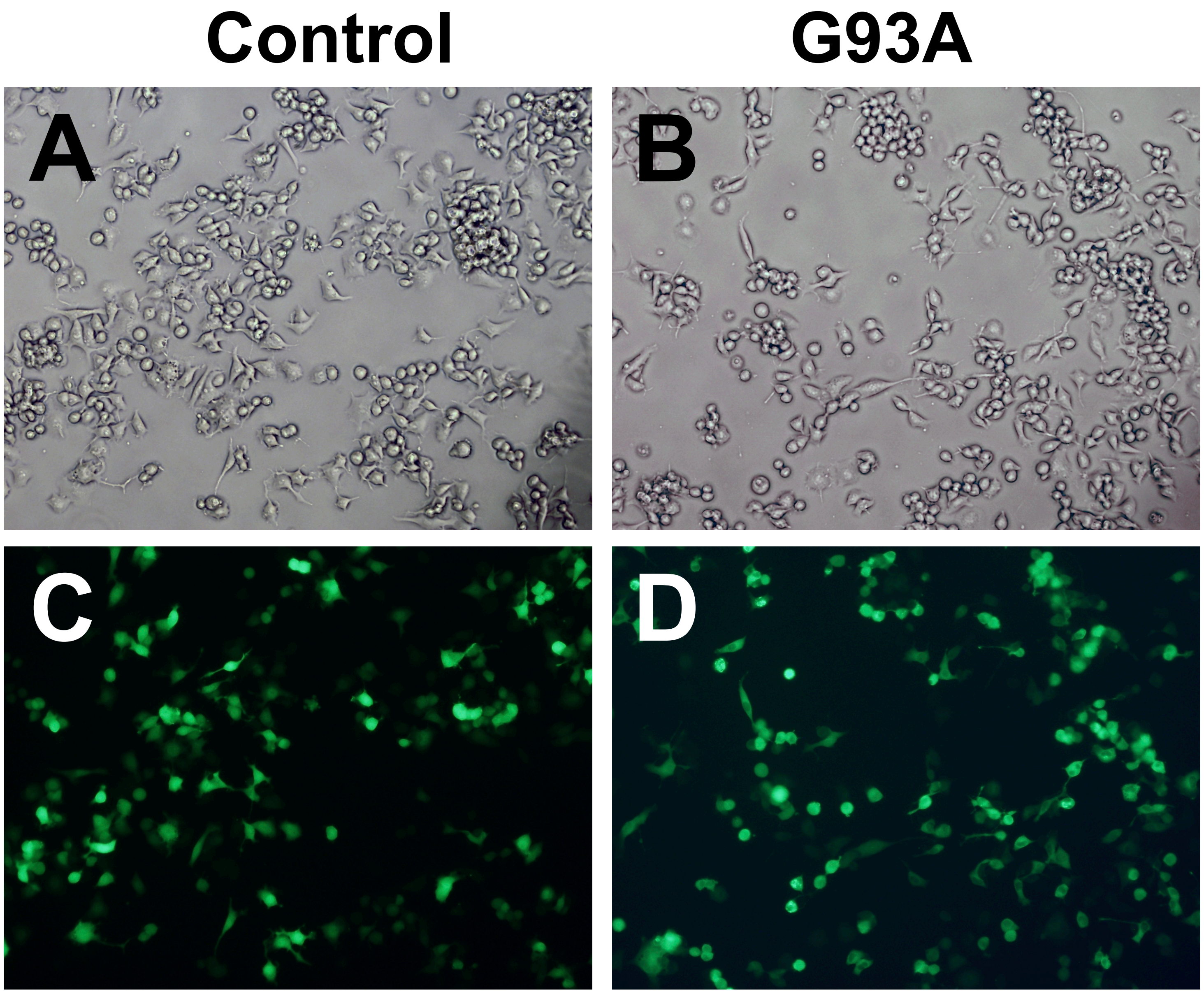

Supplement: Additional file 2: — Supplemental results.(PDF 5179 kb) [file 40478_2015_271_MOESM2_ESM.pdf]
